# Olanzapine Overdose—Induced Bradycardia in a Young Adult With Major Depressive Disorder: A Case Report and Literature Review

**DOI:** 10.1002/ccr3.71987

**Published:** 2026-02-06

**Authors:** Mohammad Ghasemi Palangi, Hassan Ahangar, Nona Sakhaie, Payman Yasami

**Affiliations:** ^1^ Department of Cardiology, School of Medicine, Ayatollah Mousavi Hospital Zanjan University of Medical Science Zanjan Iran; ^2^ School of Medicine, Ayatollah Mousavi Hospital Zanjan University of Medical Science Zanjan Iran; ^3^ Student Research Committee, Department of Cardiology, School of Medicine Zanjan University of Medical Science Zanjan Iran

**Keywords:** atypical antipsychotics, bradycardia, cardiac toxicity, case report, olanzapine overdose

## Abstract

Olanzapine, an atypical antipsychotic widely used for psychiatric disorders, is typically associated with tachycardia and QT prolongation in overdose, while bradycardia remains a rare and underrecognized effect. We report a 20‐year‐old woman with major depressive disorder who presented 4 h after ingesting 100 mg of olanzapine in a suicide attempt. She exhibited marked sinus bradycardia (41 bpm) with stable hemodynamics and no QT prolongation. Laboratory and toxicology results were unremarkable. Supportive care, including oxygen, fluids, and cardiac monitoring, led to complete recovery within 48 h. This case highlights isolated bradycardia as an uncommon but important manifestation of olanzapine toxicity. Clinicians should consider bradycardia in the cardiovascular spectrum of olanzapine overdose and ensure vigilant monitoring to prevent potential complications.

## Key Clinical Message

Olanzapine overdose can cause not only tachycardia but also rare, clinically significant bradycardia.

## 1 Introduction

Antipsychotics effectively reduce the intensity of psychotic hallucinations. First‐generation antipsychotics (FGAs) work by blocking the dopamine D2 neuron receptor [1]. Second‐generation antipsychotics (SGAs) were introduced in 1989 when researchers discovered that clozapine (Clozaril) was more effective than chlorpromazine, with fewer extrapyramidal symptoms [2]. These newer antipsychotics are considered atypical because they target neuron receptors other than dopamine. They are more effective and safer than FGAs [3]. Antipsychotics have long been known to be associated with a risk of arrhythmias and cardiac arrest. These arrhythmias often result in electrocardiogram (ECG) changes, QT prolongation, ventricular tachycardia, and torsades de pointes (TdP) [4].

Olanzapine is a thienobenzodiazepine antipsychotic approved by the FDA in 1996. It is a potent 5‐HT2 blocker with a higher 5‐HT2 than D2 occupancy at all doses [5]. Olanzapine, like other drugs (except haloperidol), increases the mean heart rate and prolongs the QT interval [6]. Adverse effects of olanzapine include extrapyramidal and anticholinergic symptoms such as weight gain, hypertriglyceridemia, and others [7, 8, 9, 10]. In overdose, patients may experience tachycardia, agitation, hyperpyrexia, ataxia, coma, and more [11]. Electrocardiographic (ECG) abnormalities may occur, including supraventricular tachycardia/arrhythmias and prolonged QTc interval, although this rarely results in torsades de pointes in retrospective analyses, possibly due to the observed mild prolongation (mean QTc 453 ± 48 ms) [12].

Bradycardia is defined as a heart rate of fewer than 60 beats per minute in adults, and while it can be normal in certain contexts, such as in athletes or during sleep, it may indicate an underlying health issue when accompanied by symptoms. Pathological causes of bradycardia include age‐related degeneration, heart disease, hypothyroidism, and electrolyte imbalances. Symptoms may manifest as dizziness, fatigue, weakness, or syncope due to inadequate blood flow to vital organs. Bradyarrhythmia encompasses a wider range of slow heart rhythms resulting from irregular electrical conduction within the heart. Unlike bradycardia, which denotes a consistently slow heart rate, bradyarrhythmias can present as variable, slow, or irregular rhythms often linked to dysfunctions in the sinoatrial (SA) or atrioventricular (AV) nodes. Examples include sinus node dysfunction and AV block, which can arise from aging, ischemic heart disease, or the effects of medications such as beta‐blockers and calcium channel blockers [13].

Although several cases of olanzapine‐associated bradycardia have been previously reported, they have largely involved elderly patients or individuals with significant comorbidities, often accompanied by hemodynamic compromise or QT prolongation. In contrast, the present report describes a young adult female who developed marked bradycardia following olanzapine overdose despite preserved hemodynamic stability and complete absence of QT interval prolongation. This distinction highlights an uncommon clinical presentation that contrasts with existing literature and underscores the importance of recognizing bradycardia as a potential manifestation of olanzapine toxicity even in otherwise healthy young patients.

This report discusses the case of a young adult female with repeated self‐poisoning episodes involving olanzapine, underscoring the need for integrated medical and psychiatric intervention.

## 2 Case History

A 20‐year‐old female with a diagnosed history of major depressive disorder presented to the emergency department (ED) with decreased consciousness. Her Glasgow Coma Scale (GCS) score was 10 upon arrival, indicating a significant reduction in alertness. The patient had ingested 20 tablets of olanzapine 5 mg (totaling 100 mg) approximately 4 h prior to her presentation, in an attempt to end her life. Before arriving at the ED, she experienced one episode of non‐bloody vomiting. She was diagnosed with major depressive disorder at age 18; the patient has had two previous suicide attempts in the last 2 years. Her medications were Sertraline 100 mg daily and Olanzapine 5 mg daily.

**FIGURE 1:**
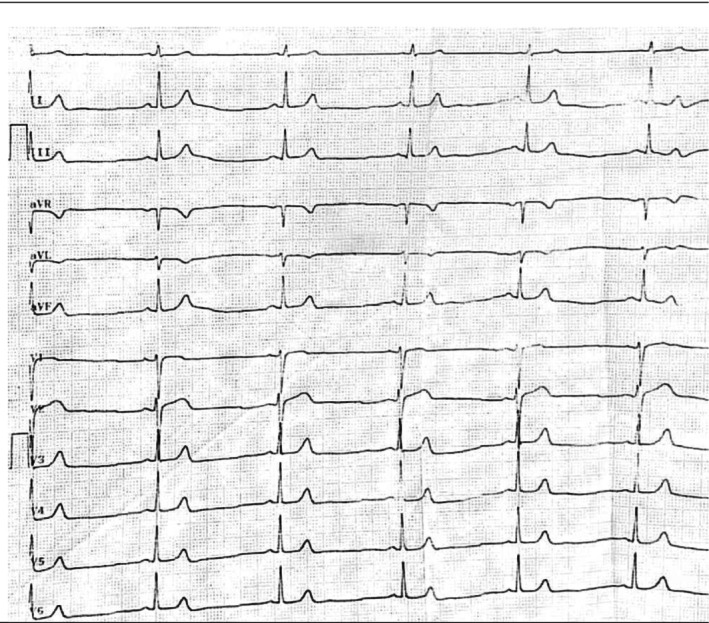
12‐lead electrocardiogram (ECG) was obtained during initial visit. The ECG was obtained in the emergency room.

In her physical examination, blood pressure was 100/60 mmHg, heart rate was 41 bpm, respiratory rate was 12 breaths/min, oxygen saturation 95% on nasal cannula, and the temperature was 36.8°C. She was unresponsive to verbal commands and responsive to painful stimuli. In the neurological examination of the patient, the pupils were 3 mm, equal and responsive to light. She showed decreased deep tendon reflexes, no focal neurological deficits, and decreased muscle tone. During heart examination, heart sounds were normal and no murmurs or extra sounds were heard. In the patient's laboratory data, liver and kidney function tests were normal, electrolytes were in the normal range, as shown in Table 1. The patient's ECG showed sinus bradycardia with QTc = 359 msec without arrhythmia (Figure 1). The toxicology screen was positive for olanzapine and negative for alcohol and other recreational drugs. The patient was admitted to the intensive care unit (ICU) for close monitoring and supportive care. Management steps include: (1) Airway and Breathing: Supplemental oxygen was provided to maintain adequate oxygen saturation. (2) Circulation: Intravenous fluids were administered to support blood pressure and hydration. (3) Cardiac Monitoring: Continuous cardiac monitoring was initiated due to the sinus bradycardia, with readiness for antiarrhythmic treatment if needed. (4) Gastrointestinal Decontamination: Activated charcoal was not administered due to the delayed presentation and decreased level of consciousness, which increased the risk of aspiration. (5) Neurological Monitoring: Frequent neurological checks were conducted to assess changes in consciousness and neurological status. (6) Psychiatric Intervention: The psychiatric team was consulted early in the management process to provide input on psychiatric stabilization and future care planning. The patient remained hemodynamically stable throughout her ICU stay. After 48 h, she regained full consciousness, and her rhythm normalized. She was then transferred to the psychiatric ward for further evaluation and management. The psychiatric team developed a comprehensive care plan involving medication review, individual psychotherapy, and family counseling. Additionally, a social worker was engaged to assess and enhance her support systems and explore community resources.

**TABLE 1:** Laboratory data.

Laboratory test	Result	Normal range
Blood glucose (BS)	98 mg/dL	70–100 mg/dL
White blood cell count (WBC)	9.1 × 10^9/L	4.0–11.0 × 10^9/L
Hemoglobin (Hb)	12.2 g/dL	13.0–17.0 g/dL (M) <br> 12.0–15.5 g/dL (F)
Hematocrit (HCT)	37.2%	38.3%–48.6% (M) <br> 35.5%–44.9% (F)
Platelet count (PLT)	218 × 10^9/L	150–450 × 10^9/L
Blood urea nitrogen (BUN)	16 mg/dL	7–20 mg/dL
Creatinine (Cr)	0.9 mg/dL	0.6–1.2 mg/dL
Sodium (Na)	142 mEq/L	135–145 mEq/L
Potassium (K)	3.7 mEq/L	3.5–5.0 mEq/L
Calcium (Ca)	9.2 mg/dL	8.5–10.2 mg/dL
Albumin (ALB)	4.5 g/dL	3.5–5.0 g/dL
Magnesium (Mg)	2.1 mg/dL	1.7–2.2 mg/dL
Phosphorus (P)	5.0 mg/dL	2.5–4.5 mg/dL
Aspartate aminotransferase (AST)	13 U/L	10–40 U/L
Alanine aminotransferase (ALT)	15 U/L	7–56 U/L
Alkaline phosphatase (ALK)	129 U/L	44–147 U/L
Total bilirubin (Bili T)	0.7 mg/dL	0.1–1.2 mg/dL
Direct bilirubin (Bili D)	0.2 mg/dL	0.0–0.3 mg/dL
Erythrocyte sedimentation rate (ESR)	7 mm/h	0–20 mm/h (M) <br > 0–30 mm/h (F)
C‐Reactive protein (CRP)	4 mg/L	< 10 mg/L
Lactate dehydrogenase (LDH)	241 U/L	125–220 U/L
Creatine phosphokinase (CPK)	88 U/L	30–223 U/L
Prothrombin time (PT)	14.6 s	11.0–13.5 s
International normalized ratio (INR)	1.1	0.9–1.1
Partial thromboplastin time (PTT)	27.4 s	25–35 s
Amylase	64 U/L	30–110 U/L
Venous blood gas (VBG)		
pH	7.37	7.35–7.45
PCO2	46.2 mmHg	35–45 mmHg
PO2	52 mmHg	75–100 mmHg
HCO3—	26.3 mEq/L	22–28 mEq/L

This patient's history of recurrent intentional overdoses underscored the need for a structured post‐discharge psychiatric follow‐up plan aimed at minimizing the risk of future self‐harm. Accordingly, scheduled outpatient psychiatric evaluations, close monitoring of medication adherence, and re‐assessment of her psychotropic regimen were arranged to ensure therapeutic safety and effectiveness. In addition, a comprehensive risk‐mitigation strategy was implemented, including means‐restriction counseling, involvement of family members in recognizing early warning signs, and coordination with community mental‐health services to support continuity of care. Such an integrated and collaborative approach is essential for patients with repeated suicide attempts, in whom ongoing psychiatric management plays a critical role in preventing recurrence.

## 3 Differential Diagnosis, Investigations, and Treatment

The differential diagnosis for the patient's marked sinus bradycardia included drug‐induced bradyarrhythmia, metabolic derangements (particularly electrolyte abnormalities), endocrine disorders such as hypothyroidism, and toxic ingestion of agents including β‐blockers, calcium channel blockers, and digoxin. Laboratory studies demonstrated normal electrolyte, renal, hepatic, and metabolic parameters, and the toxicology panel was positive only for olanzapine, narrowing the etiology to olanzapine overdose. ECG confirmed isolated sinus bradycardia (41 bpm) without QT prolongation or atrioventricular conduction defects.

Supportive treatment was initiated in the intensive care unit with supplemental oxygen, intravenous fluids, and continuous cardiac and neurological monitoring. Activated charcoal was deferred due to delayed presentation and reduced consciousness. No pharmacologic chronotropic agents were required as hemodynamics remained stable. Over the following 48 h, the patient's mental status improved and heart rhythm normalized, after which she was transferred to psychiatric services for continued evaluation and suicide risk management.

## 4 Conclusion and Results

This case report emphasizes the significant risk of bradycardia following an overdose of olanzapine, highlighting the necessity of monitoring cardiac function in patients who have consumed large doses of this medication. The occurrence of bradycardia in a patient following a suicide attempt involving olanzapine overdose illustrates the critical need for clinicians to remain alert to potential cardiovascular effects in similar situations. Timely identification and management of bradycardia are essential to avert serious complications. Additionally, this case underscores the importance of responsible prescribing practices and educating patients about the risks associated with olanzapine, particularly for those with a history of mental health disorders or suicidal behaviors. More research and case studies are necessary to further elucidate the mechanisms and risk factors linked to olanzapine‐induced bradycardia, with the goal of enhancing patient safety and clinical outcomes. By documenting this presentation, the current report contributes to the limited published descriptions of olanzapine overdose–related bradycardia and reinforces the importance of comprehensive cardiac monitoring in such cases.

## 5 Discussion

Olanzapine is an atypical antipsychotic medication widely used for the treatment of schizophrenia and bipolar disorder. It works primarily by antagonizing dopamine D2 receptors and serotonin 5‐HT2A receptors in the central nervous system, leading to improved symptoms of psychosis and mood stabilization. Its efficacy in reducing both positive and negative symptoms of schizophrenia, along with its role in managing acute manic episodes, has established olanzapine as a crucial medication in psychiatric practice [5]. In the case of overdose, olanzapine can lead to a range of adverse effects. Symptoms of overdose typically include severe sedation, respiratory depression, tachycardia, hypotension, and anticholinergic effects such as dilated pupils and dry mouth. The severity of these symptoms can vary significantly depending on the amount ingested and the presence of other co‐ingested substances, which complicates the clinical picture [12]. Olanzapine has been linked to cardiovascular adverse effects, notably QT interval prolongation and dysrhythmias. The underlying mechanism for these effects is thought to relate to olanzapine's blockade of cardiac potassium channels, particularly the human ether‐a‐go‐go‐related gene (hERG) channel, which may result in delayed repolarization and subsequent arrhythmias. Research conducted by [14] indicated that the use of olanzapine was associated with significant QT interval prolongation, heightening the risk of ventricular arrhythmias, including tachyarrhythmia and Torsades de pointes. Thus, while olanzapine is considered a valuable treatment option, it is essential for healthcare providers to remain alert to its potential to cause dysrhythmias, particularly in patients who have risk factors such as electrolyte imbalances, the concurrent use of other medications that prolong the QT interval, or existing cardiac issues [14].

The literature presents several cases of olanzapine‐induced bradycardia, highlighting significant variability in both the severity and clinical presentation of this adverse effect. These cases underscore the potential for olanzapine, even at low doses, to cause marked cardiovascular effects. For instance, Markowitz et al. reported a case of hypotension accompanied by bradycardia in a healthy volunteer following a single 5 mg dose of olanzapine. This finding is particularly important as it suggests that therapeutic doses of olanzapine may unpredictably affect heart rate in individuals without pre‐existing cardiovascular or metabolic risk factors [15]. Additionally, Rasnayake et al. described a case involving a 42‐year‐old man who experienced symptomatic bradycardia and hypothermia after the administration of olanzapine for paranoid schizophrenia. This case highlights the potential risks associated with olanzapine use in individuals with psychiatric disorders, especially when considering the interplay with comorbid symptoms such as hypothermia [16]. Mijovic et al. reported an instance of severe bradycardia (heart rate of 18 beats per minute) in a 16‐year‐old female with anorexia nervosa and sleep‐related bradycardia. This observation further indicates that vulnerable populations, including adolescents and those with metabolic or eating disorders, may exhibit heightened susceptibility to the adverse effects of olanzapine [17]. Moreover, older adults with multiple comorbidities may also be at increased risk for bradycardia. Al‐Darzi et al. documented a case involving a 65‐year‐old female with a history of schizophrenia, hypertension, and hyperlipidemia who developed sinus bradycardia, along with hypothermia and hypoglycemia. This suggests that age and comorbid conditions may exacerbate the cardiovascular response to olanzapine [18] A recent study by Sachdeva et al. further reinforces the relevance of this issue by reporting a case in which significant bradycardia developed following olanzapine use [19].

According to the literature, the cardiovascular effects associated with olanzapine predominantly highlight tachycardia and tachyarrhythmia as common manifestations, particularly in cases of overdose. This antipsychotic, primarily indicated for schizophrenia and bipolar disorder, is well‐documented to induce tachycardia due to its antagonistic effects on histamine and adrenergic receptors, which can lead to an increase in heart rate as a more typical adverse outcome. However, bradycardia and bradyarrhythmia are less frequently reported, making cases of olanzapine‐induced bradycardia a relatively rare finding in current literature.

Our case diverges from this trend, presenting a young adult with major depressive disorder who developed marked bradycardia following an olanzapine overdose. Our patient's isolated bradycardic response contrasts with the more commonly observed tachycardic effects, suggesting the need for awareness that olanzapine's cardiac effects may not always align with expected tachycardic responses and may instead present as bradycardia in certain cases. This case adds to the limited pool of olanzapine‐induced bradycardia reports, highlighting the importance of considering bradycardia as a potential but atypical response, particularly in young adults without complicating factors often present in previously reported cases (Table 2).

**TABLE 2:** Published cases of olanzapine‐induced bradycardia.

Study	Patient characteristics	Dose/Context	Key clinical findings	DOI
Markowitz et al. [15]	Healthy adult volunteer	Single 5 mg therapeutic dose	Bradycardia + hypotension	10.1177/0091270002042001013
Rasnayake et al. [16]	42‐year‐old male with paranoid schizophrenia	Therapeutic use	Symptomatic bradycardia + hypothermia	10.1186/1752‐1947‐5‐189
Mijovic et al. [17]	16‐year‐old female with anorexia nervosa	Low‐dose initiation	Severe bradycardia (*18 bpm*)	10.1093/pch/21.supp5.e60
Al‐Darzi et al. [18]	65‐year‐old woman; schizophrenia, hypertension, hyperlipidemia	Therapeutic use	Bradycardia + hypothermia + hypoglycemia	– (Conference abstract; J Gen Intern Med)
Sachdeva et al. [19]	Adult patient (case report)	Therapeutic dose	Significant bradycardia	10.4088/PCC.22cr03453

## 6 Proposed Mechanisms of Olanzapine‐Induced Bradycardia

The precise pathophysiology of olanzapine‐induced bradycardia remains incompletely understood; however, several mechanisms have been proposed:

1. *Vagal stimulation*: Enhanced parasympathetic tone may suppress sinoatrial node activity.
2. *α1‐adrenergic blockade*: Peripheral vasodilation may reduce sympathetic output and precipitate reflex bradycardia, consistent with olanzapine's receptor profile [6].
3. *Serotonergic modulation*: Through 5‐HT2A and related pathways, olanzapine may alter central autonomic regulation of cardiac rhythm. Although definitive evidence is lacking, these mechanisms align with known pharmacodynamic properties of olanzapine and may collectively contribute to bradyarrhythmia in susceptible individuals.

## 7 Comparative Contextualization with Published Cases

A comparative assessment of reported cases demonstrates substantial variability in the clinical characteristics, predisposing factors, and severity of bradycardia. Prior cases typically involved older adults, patients with psychiatric or metabolic comorbidities (e.g., anorexia nervosa), or individuals receiving therapeutic doses rather than presenting with overdose. Reported heart rate nadirs ranged from profound bradycardia (18 bpm) to milder reductions, and recovery intervals varied from several hours to multiple days. In contrast, our patient was a young adult without chronic medical illness who developed significant isolated bradycardia following an acute overdose. This deviation from the more common presentation of tachycardia in overdose scenarios reinforces the need for clinicians to recognize bradycardia as a possible, albeit atypical, manifestation of olanzapine toxicity.

## 8 Multidisciplinary and Psychosocial Considerations

Beyond acute medical management, cases involving intentional overdose warrant integrated psychosocial and rehabilitative strategies. This is especially critical for women with psychiatric disorders or functional vulnerabilities who may encounter additional barriers during crises. As emphasized in recent models of integrated care, coordinated psychiatric follow‐up, lifestyle‐focused interventions, and family and community engagement play essential roles in preventing recurrent self‐harm [20]. Incorporating multidisciplinary rehabilitation frameworks into post‐overdose care may therefore enhance long‐term outcomes by supporting both psychological and physical recovery [20].

## Author Contributions

**Mohammad Ghasemi Palangi:** conceptualization, investigation, methodology, project administration, writing – original draft, writing – review and editing. **Hassan Ahangar:** investigation, writing – original draft, writing – review and editing. **Nona Sakhaie:** investigation, methodology, writing – original draft, writing – review and editing. **Payman Yasami:** conceptualization, investigation, methodology, project administration, validation, writing – original draft, writing – review and editing.

## Funding

The authors have nothing to report.

## Consent

A written informed consent was obtained from the patient. None of the patient's identification information is presented in this manuscript.

## Conflicts of Interest

The authors declare no conflicts of interest.

## Data Availability

The data/information supporting this study is available from the corresponding author upon reasonable request.
